# Virtual educational meetings and activities during the COVID-19 pandemic and beyond: Egyptian oncologists’ experience

**DOI:** 10.3332/ecancer.2021.1275

**Published:** 2021-08-16

**Authors:** Mohamed Alorabi, Ahmed Samir Abdelhafiz, Nermen Mostafa, Asmaa Ali, Hagar Elghazawy, Ahmed Mesbah, Abdul Rahman Jazieh

**Affiliations:** 1 Department of Clinical Oncology, Faculty of Medicine, Ain Shams University, Cairo, 11591, Egypt; 2 Department of Clinical Pathology, National Cancer Institute, Cairo University, Cairo, 11792, Egypt; 3 Department of Pulmonary Medicine, Abbassia Chest Hospital, MOH, Cairo, 11517, Egypt; 4 Faculty of Medicine, Ain Shams University, Cairo, 11591, Egypt; 5 Department of Oncology, King Abdulaziz Medical City, King Saud bin Abdulaziz University for Health Sciences, King Abdullah International Medical Research Center, Riyadh, 11426, Saudi Arabia

**Keywords:** COVID-19, pandemic, continuous education, virtual meetings, oncology

## Abstract

The COVID-19 pandemic has had ramifications for most healthcare activities, including medical education and communication aspects. Virtual educational meetings and activities (VEMAs) have been utilised tremendously in the pandemic era, reflecting a transition to new horizons of cyberspace. This creates the need to explore possible challenges for the implementation of such services in the rapidly evolving field of oncology. The aim of our study is to explore the impact of the COVID-19 pandemic on VEMAs in the oncology community in Egypt. It focused on the evaluation of current attitudes, satisfaction and expectations of Egyptian oncologists during and beyond the COVID-19 era. The study is a cross-sectional study using a survey that was distributed through social media. It targeted Egyptian oncologists during the months of May and June 2020. A total of 118 participants completed the survey and most of them were younger than 35 years (71%). Most participants (93.2%) agreed that COVID-19 affected the stream of live medical educational meetings. About three-quarters of them attended VEMAs during the COVID-19 period compared to 50% prior to the pandemic. The majority reported that evening hours after 8 PM was the best time to attend VEMAs and 1 hour is the optimal duration for a virtual meeting. Although the COVID-19 pandemic appeared as an unprecedented challenge for medical education, it can be a catalyst for VEMAs, especially in a rapidly evolving field such as oncology. Further research is needed to assess whether learners are ready and willing to make greater use of online educational platforms and investigate the possible barriers and strategies to enhance their use.

## Introduction

Coronavirus disease 2019 (COVID-19) was first recognised in December 2019 in the city of Wuhan, China, and has spread to more than 200 countries in the world to date [1, 2]. The COVID-19 infection is mostly asymptomatic, but it can be fatal in 2%–3% of the cases from complications such as severe pneumonia, acute respiratory distress syndrome, septic shock and multi-organ failure [3]. The World Health Organisation (WHO) declared COVID-19 a public health emergency of international concern on 30 January and a pandemic on 11 March 2020 [4].

A few drugs and some vaccines have been approved for COVID-19. Prevention of the disease relies mainly on infection control measures such as hand washing, wearing masks, social distancing and quarantine [5, 6]. Many countries have imposed restrictions on travel in a way that has never happened before at such a scale [7]. They have also cancelled and banned mass gatherings and meetings [8].

These decisions have impacted healthcare education as many scheduled conferences, workshops and educational meetings in all fields including oncology have been cancelled, postponed or turned into a virtual format during the pandemic [9, 10]. Virtual meetings may have advantages, such as making it more convenient for more participants to attend, but on the other hand, they have drawbacks such as missing personal networking opportunities and experiencing the event first hand and in person [11].

Herein, we aim to evaluate Egyptian oncologists’ experience with virtual educational meetings and activities (VEMAs) during the COVID-19 pandemic, to correlate their use and attitude with different socio-demographic factors and to describe their needs and expectations for the post-COVID-19 era.

## Subjects and methods

### Study design and population

This cross-sectional study was conducted through an online survey for 2 months, i.e., between May and June 2020. The survey was completely anonymous. Data were collected through an online Google Forms platform. The inclusion criteria included clinical, medical, radiation and surgical oncologists practicing in the Egyptian healthcare system. The only exclusion criterion was practicing outside Egypt. The link of the survey was shared on social networking sites, including Facebook and WhatsApp groups, to the target population.

### Study tool

The survey questionnaire was designed in English and consisted of four sections. The first section covered the socio-demographic characteristics of the participants. The second section evaluated the oncologists’ attitudes towards participation in the educational activities in the year 2019, prior to the COVID-19 pandemic. Questions in the third section assessed the participants’ experience and satisfaction with the VEMAs during the COVID-19 pandemic. The last section of the survey explored their needs and expectations regarding the future of the VEMAs in the post COVID-19 world.

### Validation and pilot study

A preliminary phase was conducted to assess the validity and reliability of the questionnaire before its use. Initially, the first and the third authors, who are experts in the field of oncology and education, were asked to assess the degree to which items in the questionnaires are relevant to the goals of the study. Then, the questions were modified according to their response. After that, we tested the survey on 15 participants who completed it twice 2 weeks apart. The questionnaire had a high level of internal consistency and test-retest reliability, as determined by a Cronbach’s alpha of 0.814 and Pearson’s correlation of 0.66.

### Sampling

The sample size was determined using the Epi Info 7 software. Depending on the previous study discussing the prevalence of attendance in E-learning classes (Journal club), it reached to 5%; thus, assuming that the confidence level was 95% with a type I error of about 5%, the minimal sample size was 73 participants [12].

### Statistical analysis

The statistical analysis was carried out by Minitab 17.1.0.0 for Windows (Minitab Inc., 2013, Pennsylvania, USA). Continuous data were presented as mean and standard deviation (SD), and categorical data as number and percentage (%); the normality of data was examined using the Shapiro–Wilk test. The correlation between the attendance in VEMAs and participant characteristics was evaluated using chi-square test. The general linear model (GLM) was used to estimate the association between the different socio-demographic characteristics of the participants and the degree of agreement about VEMAs after the COVID-19 era, which is illustrated in three different questions using the Likert-type scale. All tests were two-sided, *p* was considered significant if ≤0.05.

## Results

A total of 118 oncologists had completed the survey. Table 1 depicts the socio-demographic characteristics of the participants. More than half (54.2%) of the participants were male. The mean (SD) age of participants was 34 years (+5.99); most of them (71.19%) were younger in age (≤35 years). Most of the participants (67.79%) resided and worked (72.04%) in Great Cairo governate, and about half of them (48.3%) worked in university hospitals. About 56% were clinical oncologists. The remaining were distributed equally between medical and surgical oncology (18.6%) for each specialty, and radiation oncology (6.8%). Less than half of the participants (45.8%) had a master’s degree, and more than half (55.9%) were specialists.

Table 2 illustrates the oncologists’ experience in participation in the educational activities before the COVID-19 pandemic in the year 2019, where conferences were the most frequent activity, being attended by about 90% of them. This was followed by workshops (61%), webinars (36.4%) and journal clubs (36.4%). Most of them (68.6%) presented 0–3 lectures/abstracts/posters in these activities, which were mostly inside Egypt, while about 12% of the participants had presented >10 lectures/abstracts/posters. Almost two-thirds of the participants had received funding to attend these activities. Before the COVID-19 period, half of them had experience with VEMAs and almost all of them (96%) had attended <5 educational activities outside Egypt.

Most of the participants (93.2%) agreed that COVID-19 affected the stream of live face-to-face medical educational meetings. About three-quarters of them attended VEMAs during the COVID-19 pandemic with a maximum of once or twice a week (80.7%). Smartphones and laptops (72.8% and 51.7%, respectively) were the most used devices to attend VEMAs and Zoom was the most preferred platform (85.6%) (Table 3).

The evening after 8 PM was the best time to attend VEMAs in the opinion of the majority (83.9%). The optimum frequency and duration of VEMAs were once weekly (83.05%), and for 1 hour (64.41%), respectively.

Among the advantages of VEMAs, saving more time for family and social activities ranked number one (66.9%), followed by saving travel expenses (65.3%) and the availability of free educational content (62.7%). Sixty-seven percent believed that VEMAs fulfilled their educational goals and expected learning outcomes. On the other hand, problems with Internet connections ranked number one as a barrier for participation in VEMAs (66.9%), followed by the absence of travelling experience and the absence of interaction with other participants (56.8% and 44.1%, respectively).

While more than three-quarters of the participants believed that the information gained from VEMAs can be implemented in their practice, around half of them thought that VEMAs are generally useful for their work and development.

During the COVID-19 era, attendance of VEMAs was significantly associated with clinical and medical oncology specialty (*p* < 0.001), especially consultants (*p* = 0.01) with more experience (*p* = 0.05). There was no significant impact of age, sex, qualification degree or job facility sectors on the VEMAs attendance. The correlation between socio-demographic characteristics of the participants and attendance of VEMAs during the COVID-19 pandemic is illustrated in Supplementary Table 1.

When asked about their perceptions and expectations for virtual education after the COVID-19 era, about half of the participants thought that VEMAs will grow at the expense of traditional educational activities, 73.7% hoped that international conferences will allow virtual attendance as an option for registration in the future and 61% hoped that the VEMAs will continue after the COVID-19 pandemic (Supplementary Table 2).

The correlation between socio-demographic characteristics of the participants and degree of perception about VEMAs in the post-COVID-19 world (Supplementary Table 3), quantified that females thought optimistically and hoped significantly about continuing VEMAs more than men (coefficient = 0.25 and 0.3; *p* = 0.019 and 0.02, respectively). On the other hand, doctors who work in the Ministry of Health (MOH) and private sectors showed significant disagreement about continuation of VEMAs after the COVID-19 pandemic (coefficient = −0.49 and −0.59; *p* = 0.04 and 0.02, respectively).

## Discussion

Our study revealed a significant increase in participation in VEMAs, which is a reflection of the impact of the COVID-19 pandemic on the educational activities shifted to the virtual domain.

Most participants in this survey were young oncologists ≤35 years old. About half of them were in the middle of their career, being specialists and had their master’s degrees, which is the first step for specialisation in Egypt. At this point in their career, medical doctors seek knowledge to advance their skills and to establish their career in the field, while striving to get acceptable financial revenue. However, they do not have great scientific contributions, and they are not very well known to the scientific community. This is evident from the number of lectures/abstracts/posters presented by these participants in traditional educational activities, where about two-thirds of them presented only between 0 and 3 presentations last year. Also, data reflect the fact that conference attendance increases with career stages [13], and this may explain the age group of oncologists who showed interest in this study. In two other surveys about the experience of radiation oncologists [14] and ophthalmologists [15] with virtual learning during the COVID-19 pandemic, most respondents were young physicians less than 40 years, as in our study.

Approximately 70% of the participants strongly agree that the COVID-19 pandemic affected the traditional live medical educational meetings. As a result of this, there was an increase in their participation and experience with the VEMAs in comparison to the pre-COVID-19 era (74.58% versus 50%), which means that about a quarter of the participants had this educational experience for the first time due to the current circumstances. These results are in accordance with the findings of Chatziralli *et al* [15] in their survey about the use of e-learning in ophthalmology during the COVID-19 pandemic where 48% of participating ophthalmologists from both academic and non-academic institutions worldwide did not use any e-learning before the COVID-19 but during the pandemic there was a statistically significant increase in the use of all e-learning alternatives during the pandemic.

Selection of the right time, frequency and duration of educational activities is important to ensure that the message is delivered effectively. For example, The European Molecular Biology Organisation organised a virtual conference (T6SympoZOOM) between 15 and 17 April, 2020, during the pandemic. In the post-conference survey, 131 out of 181 respondents found the conference schedule with 2-hour sessions, 3 consecutive days and 10–15 minute talks as the most appropriate for them [16]. As a result of the cancellation of all major spine educational conferences in the first half of 2020, the Virtual Global Spine Conference (VGSC) was created in April 2020 by multi-institutional board-certified and board-eligible, spine fellowship-trained neurosurgeons and a board-certified interventional neuroradiologist in the form of biweekly virtual meetings every Monday and Thursday at 1000 and 1700 Eastern Standard Time, respectively, for 60–90 minutes [17]. In our study and from the answers, we can say that most of the participants prefer attending a maximum of one or two VEMAs per week, and in their opinion the most appropriate time to hold them is after 8 PM. Perhaps, they prefer this time because the majority would have finished their work, returned to home and had some rest before attending any VEMAs. Also, learning before night-time sleep has been shown to foster the consolidation of new memories [18], which may represent another advantage of this timing. Regarding the duration of the VEMAs, most participants believe it should be an hour or less. This is consistent with the fact that typical learner attention span wanes after about 15–20 minutes [19]. After 20 minutes, lectures become less effective for two reasons: working memory and interference, which make longer lectures both less enjoyable and less effective [19, 20]. Taken together, we think that scientific societies should take the perspectives of the audience into consideration while planning for their VEMAs. They should also be aware that delivering their content online at their homes does not mean the target audience will always be available to receive this content.

The affordable and easy access to smartphones in everyday social and work life of participants made them the preferred tool to be used in the VEMAs (72.9%). In one of the studies conducted on university students, it was found that 66% use mobile devices for e-learning, 45% used a mobile phone and 38% a laptop [21].

Zoom application was the preferred platform in our study to conduct VEMAs. Although not completely free, the application became very popular during social distancing time, recruiting millions of users and achieving great profits [22]. The application was the preferred platform for e-learning during the COVID-19 pandemic in many other studies [15–17, 23–25].

Education comes with a cost. Although free educational courses are now available online on multiple platforms, international conferences and workshops are expensive especially for early and mid-career physicians in developing countries [26]. Salaries for physicians in Egypt are among the lowest in the region [27]. That is why it is important for these physicians to get financial support to attend these meetings to cover the registration and travel costs. This was clear in the answers of our participants, where the great majority of them depended on external fund sources to attend educational activities. However, we did not ask about the source of this fund (whether it comes from their work place or pharmaceutical companies). This cost is usually obviated during VEMAs, which explains why participants considered that the major advantage of virtual education is saving money through the availability of free content. A Norwegian study about the benefit of 88 learners from online education in dermatology found that the participants saved 455,198 Norwegian krone in travel expenses [28], which is equivalent to 81,189 US dollars according to the average exchange rate in 2011 [29]. In another study about the impact of a Small private online course (SPOC) in oncology education and after comparison of the overall cost of the SPOC and the theoretical costs of comparable face-to-face education, the SPOC was a more cost-effective educational method (€148,000, versus €154,000 for face-to-face education) [30]. Although the fact that virtual conferences are supposed to consume lesser budget than needed by live conferences, still some landmark oncology conferences amid the COVID-19 pandemic require registration fees that reach 300$/participant [31]. The results of our survey guided the oncology community to exert their efforts to try to provide the VEMAs with amenable charges that can fit the lower income countries.

Burnout is common among oncologists including Egyptians as they have a stressful career [32–34]. The relationship with family members and practicing activities and hobbies are effective in reducing stress and overcoming burnout [35]. Flexible time management and saving travel time were advantages for online education according to students attending electrocardiogram course at the University of Ulm [36]. In this survey, it seems that social distancing, limited travel and VEMAs enabled our participants to save more time for family/activities. We think that this represents the other side of the coin, where physicians who were forced to ‘stay at home’ could regain their social life partially.

However, VEMAs lack some advantages which are provided by live events. Conferences and live educational meetings represent good opportunities for attendees to interact with their peers in the field as reported in a survey of travel behaviour among scientists in Germany. This is particularly important for early career doctors and scientists, where they can also have a chance to know about different cultures [13, 37]. A systematic review of the factors affecting e-learning in health sciences found that lack of interaction and collaboration between learners and facilitators is a barrier for this type of education [38]. This was evident in the responses of our participants, who reported the lack of interactions with other participants and experience with new cultures among the drawbacks of VEMAs.

Internet connection speed in Egypt improved in 2020 to rank number no. 2 in Africa, in comparison to 2017 [39], where Egypt’s Internet speed ranked among the world’s slowest [40]. However, a major problem with VM in this survey was the problem with Internet connection speed in Egypt, which indicates that major efforts should be carried out to overcome this problem and improve the speed. It seems that the problem with Internet connection and its speed is an obstacle to education in other countries as well. Healthcare worker in sub-Saharan Africa who completed the University of Washington School of Medicine online graduate course reported slow or limited Internet among the barriers to course completion [41]. However, in a survey about the online education for undergraduate health professional in Jordan during the COVID-19 pandemic, the lack of past experiences on using online tools was identified as the main barrier to e-learning among 75% of the participants in comparison to 13% in our study [42]. This difference may be due to the difference in age and experience between participants in the two studies.

A great majority of our participants believed that the information learnt from VEMAs can be implemented in their practice. This is similar to the results proved by other healthcare professionals, where distance learning was found to be effective for oncology nurses and neurosurgeons training [43, 44]. However, a fewer number of participants reported that VEMAs are useful for their work and development, which may reflect that the content should be more tailored to match their needs.

Certainly, the world will change after this pandemic in different aspects, including education with fears of a new recession and financial collapse [45]. VEMAs need less costs and arrangements. This may be why participants think and hope the VEMAs will continue and grow at the expense of traditional educational activities after the COVID-19 pandemic. These opinions and expectations are consistent with the opinions of other healthcare professionals, including neurosurgeons and orthopaedists, who intend to participate in e-learning activities after the end of this pandemic [43, 46]. Our respondents also hope international conferences to put virtual attendance as an option for registration in the future.

In the present study, the attendance of VEMAs during the COVID-19 era was significantly associated with the clinical and medical specialty (*p* < 0.001), especially consultants (*p* = 0.01) with more experience (*p* = 0.05), mostly because most participants were clinical or medical oncologists. Having more senior physicians attending these activities may reflect a targeted marketing to attract this particular group, or the ability to manage their time better to attend the meeting or being more experience in identifying these events and connecting with them. Around 1,000 Libyan doctors participated in a survey about the interaction of pharmaceutical company representative with doctors. There was a statistically significant difference in visiting rates based on the age group and all doctors older than 45 years who communicated with pharmaceutical company representatives [47]. Likewise, continuous medical education participation was more common among older Chinese doctors with intermediate or senior job titles in a nationwide cross-sectional survey [48].

E-learning may be more favourable for women through its flexible and interactive learning approach [49]. Interestingly, female participants in our study thought optimistically and hoped that VEMAs will continue more than male participants. Young female physicians often experience difficulty in returning to a clinical setting once they start having children and a Japanese nationwide cohort revealed that the proportion of female physicians who take leave is higher than the proportion of those who return to work [50]. We think that VEMAs are convenient for female doctors and may help them to achieve a balance between updating their knowledge continuously and caring for their homes and families. On the other hand, MOH and private sectors doctors were less optimistic about this point. This may reflect their need for more networking with their peers in other universities and with international experts as well.

The proposed strategies that could encourage oncologists to engage in future VEMAs include using attractive platforms for conferences as for instance the 3D interactive virtual platform and organising the VEMAs schedules to avoid overlapping. Moreover, CME accreditation and offering the lectures in recorded forms to be retrieved in the convenient time will up skill the oncologists’ carrier in real life. On the other hand, providing the chance for the junior oncologists to directly reach and get in touch with the oncology international experts and mentees in different fields virtually can greatly motivate them to attend these events and overcome financial obstacles for oversees collaborations and training. Finally, providing post-meeting satisfaction surveys for the speakers and attendees will help in evaluating and improving these services and upgrade its content.

To the best of our knowledge, there is no published data yet on the evaluation of the impact of the COVID-19 pandemic on virtual educational practices in the oncology field in Egypt or Arab countries. This research aims to explore the socio-demographic attributes of oncologists’ willingness to achieve virtual education, identifying the possible barriers for participation in these activities and clarify their needs for future events. In an attempt to improve the implementation of the VEMAs in the post-COVID-19 era, this study will enhance the technical skills of oncologists to use the virtual platforms in order to maximise the benefits of attending such meetings.

This study illustrated several important results by taking a snapshot for the current perspective towards VEMAs amid the COVID-19 pandemic. However, some limitations should be considered. Firstly, this survey was conducted and administered through social networking sites, including Facebook and WhatsApp groups, and this may result in sampling bias, e.g., most participants were young as only those who were interested and familiar with social media answered the survey. Secondly, the cross-sectional design of this study. Thirdly, limited sample size was among the study limitations. Fourthly, we did not evaluate the speakers or moderators’ experience as they may have different perspectives or needs. Fifth, we did not ask about hybrid meetings since they were not common options at the time of the distribution of the survey. Finally, and importantly, the participants’ perspective regarding the curriculum or the content of VEMAs was not deeply explored. VEMAs may represent a golden opportunity to exchange knowledge and experience with experts in the fields and follow the latest updates, but it is difficult to learn manual skills through them or utilise them when direct interaction with patients is needed. So, it is not possible to generalise this type of educational activities to all specialties and fields, which mandates a separate evaluation for other disciplines.

## Conclusion and recommendations

The COVID-19 pandemic has led to new opportunities for virtual education. Curricula for this type of education should be developed and tailored according to the needs of target groups. Virtual registration and free content should be added as an option in future international scientific events to help young physicians in developing countries improve their knowledge and skills, without the need to bear the costs for travel and accommodation.

## Conflicts of interest

The authors declare that they have no conflicts of interest.

## Funding source

No source of external funding was received for this research.

## Figures and Tables

**Supplementary Table 1.** Correlation between socio-demographic characteristics of the participants and attendance of virtual educational meetings and activities during the COVID-19 pandemic.

Socio-demographic characteristics	No virtual meeting/activity attendance (*n* = 30)		Attending virtual meeting/activity (*n* = 88)		*p*
Age	32.73	4.86	34.68	6.27	0.08^a^
**Age groups**					
Young (≤35 years)	25	83.33	59	67.05	
Old (> 35 years)	5	16.67	29	32.95	0.08^b^
**Sex**					
Female	10	33.33	44	50	0.11^b^
Male	20	66.67	44	50	
**Specialty**					
Clinical oncology	9	30	57	64.77	<0.001^b^
Medical oncology	4	13.33	18	20.45	
Radiation oncology	5	16.67	3	3.41	
Surgical oncology	12	40	10	11.36	
Years of experience	6.77	5.07	8.97	6.02	0.05^a^
**Highest medical degree**					
PhD/MD	4	13.33	30	34.09	0.08^b^
MSc	19	63.33	35	39.77	
Diploma	2	6.67	2	2.27	
MBBCh	4	13.33	16	18.18	
Others	1	3.33	5	5.68	
**Current job title**					
Consultant	2	6.67	27	30.68	0.01^b^
Specialist	21	70	45	51.14	
Resident	7	23.33	16	18.18	
**Medical facility**					
University Hospital	11	36.67	46	52.27	0.1^b^
Ministry of Health Hospital	5	16.67	19	21.59	
Military/Police Hospital	5	16.67	10	11.36	
Private Hospital/Clinic	4	13.33	10	11.36	
Others	5	16.67	3	3.41	

a Independent *t*-test

b Chi-square test

*p* considered significant if <0.05

**Supplementary Table 2.** Needs and expectations for the future of virtual educational meetings and activities in the post-COVID-19 world (*n* = 118).

Items	No	%
**After the COVID-19 pandemic, do you think virtual educational meetings and activities will grow at the expense of traditional educational activities**		
Yes	58	49.15
No	20	16.95
Not sure	40	33.9
**I think the virtual educational meetings and activities will continue after the COVID-19 pandemic**		
Strongly agree	10	8.47
Agree	51	43.22
Neutral	34	28.81
Disagree	13	11.02
Strongly disagree	10	8.47
**I hope the virtual educational meetings and activities will continue after the COVID-19 pandemic**		
Strongly agree	23	19.49
Agree	50	42.37
Neutral	21	17.8
Disagree	15	12.71
Strongly disagree	9	7.63
**I hope the international conferences will allow virtual attendance as an option for registration in the future**		
Strongly agree	44	37.29
Agree	43	36.44
Neutral	15	12.71
Disagree	7	5.93
Strongly disagree	9	7.63

**Supplementary Table 3.** Correlation between the socio-demographic characteristics of participants and the degree of perception about virtual education after the COVID-19 pandemic.

Socio-demographic characteristics	I think the virtual educational meetings and activities will continue after COVID-19 pandemic		I hope the virtual educational meetings and activities will continue after COVID-19 pandemic		I hope international conferences will allow virtual attendance as an option for registration in the future	
	Coeff	*p*	Coeff	*p*	Coeff	*p*
**Age groups**						
Young (≤35 years)	−0.105	0.442	−0.04	0.804	−0.081	0.627
Old (> 35 years)	Ref					
**Sex**						
Female	0.257	0.019	0.3	0.021	0.191	0.147
Male	Ref					
**Specialty**						
Clinical oncology	−0.309	0.066	−0.007	0.971	−0.073	0.72
Medical oncology	−0.181	0.422	−0.174	0.512	−0.158	0.562
Radiation oncology	0.192	0.535	−0.029	0.936	0.254	0.5
Surgical oncology	Ref					
**Highest medical degree**						
PhD/MD	Ref					
MSc	0.223	0.324	0.02	0.941	0.491	0.075
Diploma	−0.26	0.58	0.108	0.846	−0.531	0.352
MBBCh	0.109	0.677	0.26	0.401	0.476	0.135
Others	−0.166	0.657	−0.227	0.608	−0.473	0.298
**Current job title**						
Consultant	−0.027	0.929	0.196	0.583	0.135	0.713
Specialist	Ref					
Resident	0.4	0.123	0.001	0.996	0.084	0.789
**Medical facility**						
University Hospital	Ref					
Ministry of Health Hospital	−0.232	0.267	−0.499	0.044	−0.187	0.46
Military/Police Hospital	0.355	0.145	0.458	0.112	0.006	0.984
Private Hospital/Clinic	−0.596	0.021	−0.059	0.844	−0.304	0.326
Others	0.72	0.024	0.358	0.337	0.592	0.124

GLM: General linear model, Co-eff: coefficient

*p* considered significant if <0.05

**Table 1.** Socio-demographic characteristics of the participants (*n* = 118).

Socio-demographic characteristics	No	%
Age (mean ±SD)	34.19	5.99
**Age groups**		
≤35 years	84	71.19
> 35 years	34	28.81
**Sex**		
Female	54	45.76
Male	64	54.24
**Residence**		
Great Cairo	80	67.79
Alexandria	10	8.47
Delta	12	10.16
Canal	8	6.79
Upper Egypt	8	6.79
**Workplace**		
Great Cairo	85	72.04
Alexandria	5	4.24
Delta	12	10.16
Canal	3	2.54
Upper Egypt	13	11.02
**Specialty**		
Clinical oncology	66	55.93
Medical oncology	22	18.64
Radiation oncology	8	6.78
Surgical oncology	22	18.64
Years of experience (mean ± SD)	8.41	5.85
**Highest medical degree**		
PhD/MD	34	28.81
MSc	54	45.76
Diploma	4	3.39
MBBCh	20	16.95
Others	6	5.08
**Current job title**		
Consultant	29	24.58
Specialist	66	55.93
Resident	23	19.49
**Medical facility**		
University Hospital	57	48.31
Ministry of Health Hospital	24	20.34
Military/Police Hospital	15	12.71
Private Hospital/Clinic	14	11.86
Others	8	6.78

**Abbreviations:** Ph.D./MD: Doctor of Philosophy/ Doctor of Medicine, MSc: Master of Science**,** MBBCh: Bachelor of Medicine, Bachelor of Surgery

**Table 2.** Participation in the educational activities before the COVID-19 era (*n* = 118).

Items	No	%
**Attended educational activities**		
Conferences	106	89.83
Journal clubs	43	36.44
Seminars	36	30.51
Webinars	43	36.44
Workshops	72	61.02
**Number of presented lectures/abstracts/posters in these activities per participant**		
0–3	81	68.64
3–5	17	14.41
5–10	6	5.08
More than 10	14	11.86
**Number of attended educational activities inside Egypt per participant**		
0–5	58	49.15
5–10	44	37.29
10–20	9	7.63
More than 20	7	5.93
**Number of attended educational activities outside Egypt per participant**		
0–5	113	95.76
5–10	5	4.24
**Receiving a fund to attend educational activities**		
Yes (Sponsored)	54	45.76
No (Self-funded)	33	27.97
Both	31	26.27
**Experience with virtual meeting**		
Yes	59	50
No	59	50

**Table 3.** Virtual educational meetings and activities (VEMAs) experience and satisfaction during the COVID-19 pandemic era (*n* = 118).

Items	No	%
**I think that the COVID-19 pandemic affected live medical educational meetings**		
Strongly agree Agree Neutral Disagree Strongly disagree	82 28 6 2 6	69.49 23.73 5.08 1.69 5.08
**Did you attend any virtual educational meetings and activities during the COVID-19 pandemic?**		
Yes	88	74.58
No	30	25.42
**If the answer is yes, how many per week?**		
(1–2)	71	80.68
(2–5)	23	19.32
**What is your preferred video conference platform for virtual educational meetings and activities** ^a^		
GoToMeeting	14	11.86
Microsoft Teams	25	21.19
A platform of pharmaceutical company	10	8.47
Skype	10	8.47
Zoom	101	85.59
Others	7	5.93
**What are the devices used in the virtual educational meetings and activities** ^a^		
Desktop computers	5	4.24
Laptop computers	61	51.69
Smartphones	86	72.88
Tablets	22	18.64
**In your opinion, what time of the day is most convenient to attend a live Virtual Event?**		
(6–11) AM	3	2.54
(11AM–4PM)	7	5.93
(4–8) PM	9	7.63
After 8 PM	99	83.9
**In your opinion, how long should a virtual medical educational meeting/activity last?**		
Less than an hour	28	23.73
Hour	76	64.41
More than an hour	14	11.86
**In your opinion, what is the accepted frequency of virtual educational meetings and activities?**		
Daily	2	1.69
Weekly	98	83.05
Monthly	15	12.71
Others	3	2.54
**In your opinion, what are the benefits of virtual educational meetings and activities?** ^a^		
Avoid the cost of travel	77	65.25
Avoid problems with getting a visa	54	45.76
On-demand content at my convenience	52	44.07
Saving more time for my family/activities	79	66.95
Saving more time for work/research	55	46.61
Free content that is available for everyone	74	62.71
Others	6	5.08
**In your opinion, what are the problems and disadvantages of virtual educational meetings and activities?** ^a^		
No interactions with participants	52	44.07
Inconvenience with timing	37	31.36
No social networking	35	29.66
No travelling or experience with new cultures	67	56.78
Problems with internet connections	79	66.95
Technically difficult to use	16	13.56
Others	8	6.78
**Virtual educational meetings and activities fulfil your educational goals and expected learning outcomes**		
Strongly agree	21	17.8
Agree	59	50
Neutral	32	27.12
Disagree	6	5.08
**The information you learnt from virtual educational meetings and activities can be implemented in your practice**		
Strongly agree	29	24.58
Agree	62	52.54
Neutral	24	20.34
Disagree Strongly Disagree	2	1.7
	1	0.8
**Virtual educational meetings and activities are useful for your work and development**		
Strongly agree	7	5.93
Agree	51	43.22
Neutral	30	25.42
Disagree	6	5.08
Strongly disagree	24	20.34

a Participants were allowed to choose more than one answer.

## References

[1] Lee A (2020). Wuhan novel coronavirus [COVID-19]: why global control is challenging?. Public Health.

[2] Sun P, Lu X, Xu C (2020). Understanding of COVID-19 based on current evidence. J Med Virol.

[3] Guan WJ, Ni ZY, Hu Y (2020). Clinical characteristics of coronavirus disease 2019 in China. N Engl J Med.

[4] Shi D, Lu H, Wang H (2020). A simulation training course for family medicine residents in China managing COVID-19. Aust J Gen Pract.

[5] Rismanbaf A (2020). Potential treatments for COVID-19; a narrative literature review. Arch Acad Emerg Med.

[6] Abdelhafiz AS, Mohammed Z, Ibrahim ME (2020). Knowledge, perceptions, and attitude of Egyptians towards the novel coronavirus disease [COVID-19]. J Community Health.

[7] Devi S (2020). Travel restrictions hampering COVID-19 response. Lancet.

[8] Güner R, Hasanoğlu I, Aktaş F (2020). COVID-19: Prevention and control measures in community. Turk J Med Sci.

[9] Almarzooq ZI, Lopes M, Kochar A (2020). Virtual learning during the COVID-19 pandemic: a disruptive technology in graduate medical education. J Am Coll Cardiol.

[10] Kalia V, Srinivasan A, Wilkins L (2020). Adapting scientific conferences to the realities imposed by COVID-19. Radiol Imaging Cancer.

[11] Killock D (2020). From the ASCO20 virtual meeting. Nat Rev Clin Oncol.

[12] Rosewall T (2012). The use of journal clubs in canadian radiation therapy departments: prevalence and perceptions. J Med Imaging Radiat Sci.

[13] Haage V (2020). A survey of travel behaviour among scientists in Germany and the potential for change. Elife.

[14] McDowell L, Goode S, Sundaresan P (2020). Adapting to a global pandemic through live virtual delivery of a cancer collaborative trial group conference: the TROG 2020 experience. J Med Imaging Radiat Oncol.

[15] Chatziralli I, Ventura CV, Touhami S (2020). Transforming ophthalmic education into virtual learning during COVID-19 pandemic: a global perspective. Eye (Lond).

[16] Salomon D, Feldman MF (2020). The future of conferences, today: are virtual conferences a viable supplement to ‘live’ conferences?. EMBO Rep.

[17] Rasouli JJ, Shin JH, Than KD (2020). Virtual spine: a novel, international teleconferencing program developed to increase the accessibility of spine education during the COVID-19 pandemic. World Neurosurg.

[18] Holz J, Piosczyk H, Landmann N (2012). The timing of learning before night-time sleep differentially affects declarative and procedural long-term memory consolidation in adolescents. PloS One.

[19] Cooper AZ, Richards JB (2017). Lectures for adult learners: breaking old habits in graduate medical education. Am J Med.

[20] Huggett KN, Jeffries WB (2014). An Introduction to Medical Teaching.

[21] Neil R, Michael R (2014). Student use of mobile devices in university lectures. Australas J Educ Technol.

[22] Evans D How zoom became so popular during social distancing 2020. https://www.cnbc.com/2020/04/03/how-zoom-rose-to-the-top-during-the-coronavirus-pandemic.html.

[23] Odedra D, Chahal BS, Patlas MN (2020). Impact of COVID-19 on Canadian radiology residency training programs. Can Assoc Radiol J.

[24] O’Connell A, Tomaselli PJ, Stobart-Gallagher M (2020). Effective use of virtual gamification during COVID-19 to deliver the OB-GYN core curriculum in an emergency medicine resident conference. Cureus.

[25] Figueroa F, Figueroa D, Calvo-Mena R (2020). Orthopedic surgery residents’ perception of online education in their programs during the COVID-19 pandemic: should it be maintained after the crisis?. Acta Orthop.

[26] Arend ME, Bruijns SR (2019). Disparity in conference registration cost for delegates from low- and middle-income backgrounds. Afr J Emerg Med.

[27] Soliman SSA, Hopayian K (2019). Egypt: on the brink of universal family medicine. Br J Gen Pract.

[28] Schopf T, Flytkjær V (2011). Doctors and nurses benefit from interprofessional online education in dermatology. BMC Med Educ.

[29] https://www.exchangerates.org.uk/USD-NOK-spot-exchange-rates-history-2011.html.

[30] Vaysse C, Chantalat E, Beyne-Rauzy O (2018). The impact of a small private online course as a new approach to teaching oncology: development and evaluation. JMIR Med Educ.

[31] ASCO https://meetings.asco.org/am/registration.

[32] Allegra CJ, Hall R, Yothers G (2005). Prevalence of burnout in the US Oncology community: results of a 2003 survey. J Oncol Pract.

[33] Banerjee S, Califano R, Corral J (2017). Professional burnout in European young oncologists: results of the European Society for Medical Oncology [ESMO] Young Oncologists Committee Burnout Survey. Ann Oncol.

[34] Alorabi M, Ghali R, Boulos D (2015). 1546 cross-sectional study of burnout among a group of Egyptian oncologists at Ain Shams University. Eur J Cancer.

[35] Nowakowski J, Borowiec G, Zwierz I (2017). Stress in an oncologist’s life: present but not insurmountable (Students should not be discouraged choosing oncology as their future specialisation). J Cancer Educ.

[36] Keis O, Grab C, Schneider A (2017). Online or face-to-face instruction? A qualitative study on the electrocardiogram course at the University of Ulm to examine why students choose a particular format. BMC Med Educ.

[37] Mishra S (2016). Do medical conferences have a role to play? Sharpen the saw. Indian Heart J.

[38] Regmi K, Jones L (2020). A systematic review of the factors – enablers and barriers – affecting e-learning in health sciences education. BMC Med Educ.

[39] Refaat T (2020). Egypt Ranks 2nd in Africa in terms of Internet speed. https://see.news/egypt-ranks-2nd-in-africa-in-terms-of-internet-speed/.

[40] Noureldin O (2020). Egypt’s internet speed among world’s slowest, ranking 146 out of 150. https://egyptindependent.com/egypt-s-internet-speed-among-world-s-slowest-ranking-146-out-150/.

[41] Feldacker C, Jacob S, Chung MH (2017). Experiences and perceptions of online continuing professional development among clinicians in sub-Saharan Africa. Hum Resour Health.

[42] Muflih S, Abuhammad S, Karasneh R (2020). Online Education for Undergraduate Health Professional Education during the COVID-19 Pandemic: Attitudes, Barriers, and Ethical Issues.

[43] Downes SR, Lykina T (2020). Closing the gap in global neurosurgical education via online conference: a pre-covid survey. Cureus.

[44] das Graças Silva Matsubara M, De Domenico EBL (2016). Virtual learning environment in continuing education for nursing in oncology: an experimental study. J Cancer Educ.

[45] Nicola M, Alsafi Z, Sohrabi C (2020). The socio-economic implications of the coronavirus pandemic [COVID-19]: a review. Int J Surg.

[46] Figueroa F, Figueroa D, Calvo-Mena R (2020). Orthopedic surgery residents’ perception of online education in their programs during the COVID-19 pandemic: should it be maintained after the crisis?. Acta Orthop.

[47] Alssageer MA, Kowalski SR (2012). A survey of pharmaceutical company representative interactions with doctors in Libya. Libyan J Med.

[48] Wong WCW, Zhu S, Ong JJ (2017). Primary care workforce and continuous medical education in China: lessons to learn from a nationwide cross-sectional survey. BMJ Open.

[49] Bruestle P, Haubner D, Schinzel B (2009). Doing e-learning/doing gender? Examining the Relationship between Students’ Gender Concepts and E-learning Technology.

[50] Kodama T, Koike S, Matsumoto S (2012). The working status of Japanese female physicians by area of practice: cohort analysis of taking leave, returning to work, and changing specialties from 1984 to 2004. Health policy.

